# Supplementary material for: Chemical Composition, Larvicidal and Molluscicidal Activity of Essential Oils of Six Guava Cultivars Grown in Vietnam
Source: Plants (Basel). 2023 Aug 7;12(15):2888. doi: 10.3390/plants12152888 (PMC10421063; doi:10.3390/plants12152888)
Supplement: Supplementary file 1 [file plants-12-02888-s001.zip › Supplementary Materials, Figure S1.pdf]

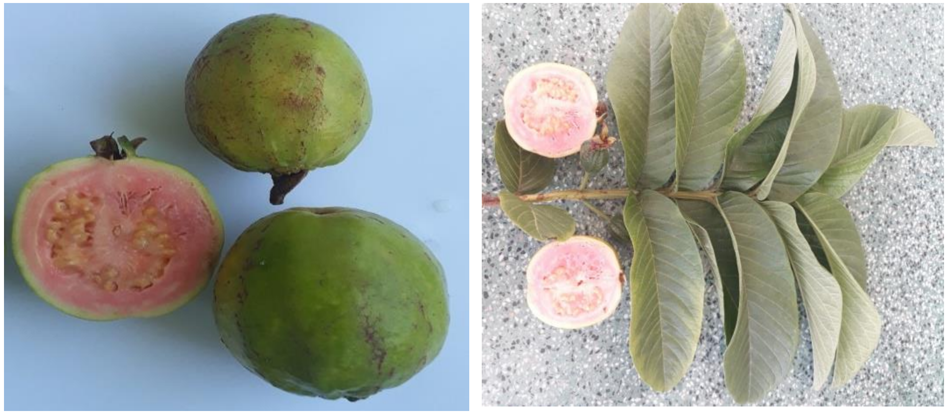

Fruit and leavesof cultivars Pink Pearl Guava (Code PG01)

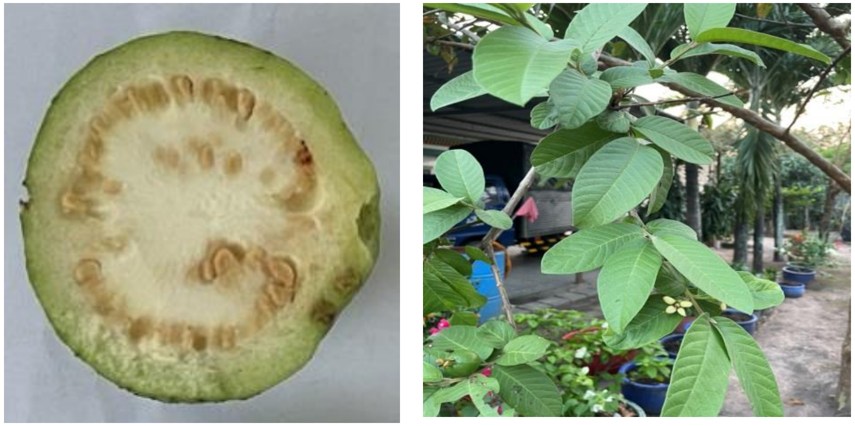

Fruit and leaves of cultivars White flesh Guava (Code PG02)

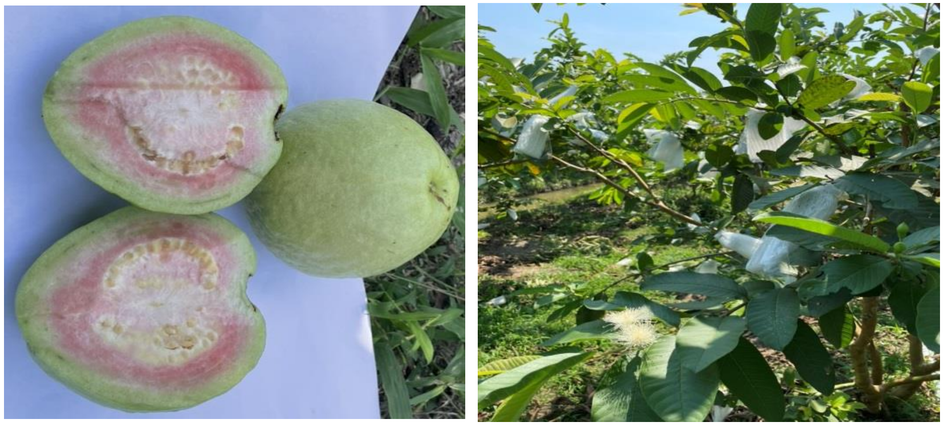

Fruit and leaves of cultivars Pink flesh smooth skin Guava (Code PG03)

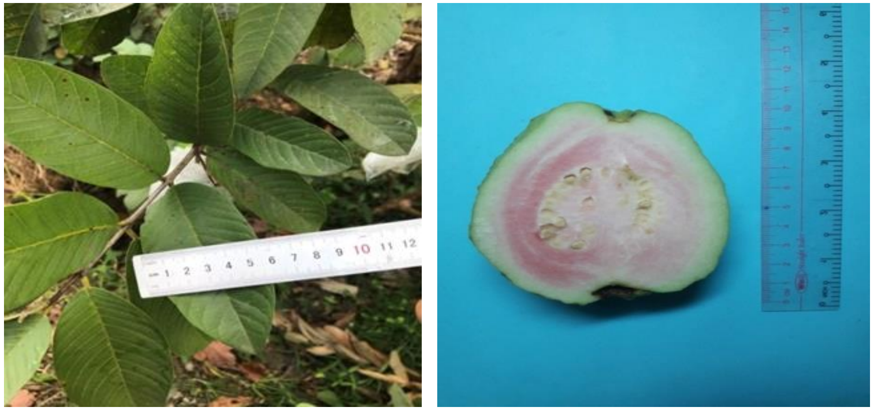

Leaves and fruit of cultivars Pink flesh rough skin Guava (Code PG04)

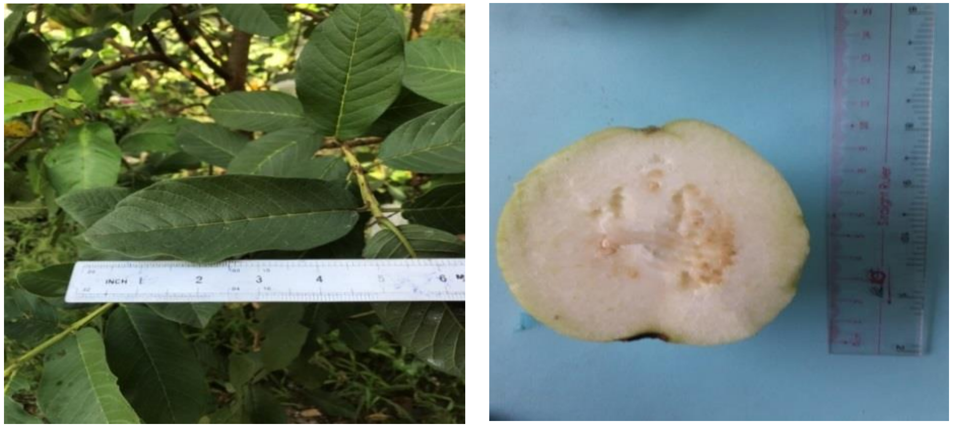

Leaves and fruit of cultivars Taiwan Guava (Code PG05)

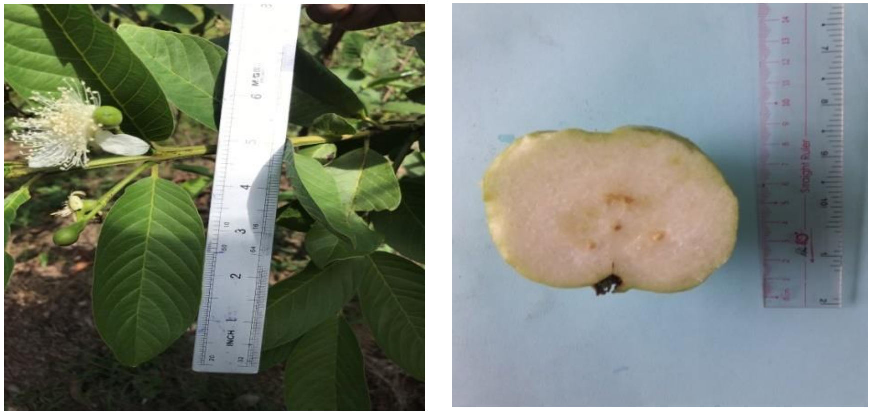

Leaves and fruit of cultivars Queen Guava (Code PG06)
