# Supplementary material for: Chemical Composition, Larvicidal and Molluscicidal Activity of Essential Oils of Six Guava Cultivars Grown in Vietnam
Source: Plants (Basel). 2023 Aug 7;12(15):2888. doi: 10.3390/plants12152888 (PMC10421063; doi:10.3390/plants12152888)
Supplement: Supplementary file 1 [file plants-12-02888-s001.zip › Supplementary Materials, Table S1.pdf]

Chemical composition of essential oils of six varieties of guava in Vietnam

RI(calc)	RI(db)	Compound	VL-41	VL-42	VL-43	VL-44	VL-45	VL-46
920	919	Hashishene	tr	tr	tr	-	-	tr
925	925	α-Thujene	tr	tr	tr	-	-	tr
933	933	**α Pinene**	**13.0**	**0.5**	**0.4**	**tr**	**0.3**	**0.1**
947	948	α-Fenchene	tr	-	tr	-	-	-
949	950	Camphene	0.1	tr	tr	-	-	-
952	953	Thuja-2,4(10)diene	tr	-	-	-	-	-
960	960	Benzaldehyde	tr	tr	tr	-	tr	tr
977	978	β-Pinene	0.2	0.1	tr	tr	0.1	tr
988	989	Myrcene	0.2	0.5	0.2	tr	0.4	0.1
1004	1004	*p*-Mentha-1(7),8-diene	-	0.1	-	-	tr	-
1004	1005	(3*Z*)-Hexenyl acetate	-	-	tr	-	-	tr
1005	1006	3-Ethenyl-1,2-dimethylcyclohexa-1,4-diene	-	tr	-	-	-	-
1006	1006	α-Phellandrene	-	tr	tr	-	0.1	tr
1011	1012	Hexyl acetate	-	-	tr	-	-	tr
1016	1017	α-Terpinene	-	tr	-	-	tr	tr
1024	1024	*p*-Cymene	0.1	0.2	0.3	tr	0.1	tr
1029	1030	**Limonene**	**0.7**	**26.2**	**1.3**	**0.4**	**20.8**	**0.5**
1030	1031	β-Phellandrene	tr	0.1	0.1	tr	0.1	tr
1031	1032	1,8-Cineole	0.2	1.0	1.1	tr	0.6	0.1
1034	1034	(*Z*)-β-Ocimene	0.4	0.3	0.8	0.2	0.1	0.6
1045	1045	(*E*)-β-Ocimene	0.1	0.1	0.2	0.1	0.1	0.2
1057	1057	γ-Terpinene	-	0.1	tr	tr	0.2	tr
1063	1064	Prenyl butyrate	tr	-	-	-	-	-
1085	1086	Terpinolene	tr	tr	tr	tr	tr	tr
1090	1091	Rosefuran	tr	tr	tr	-	-	-
1094	1097	α-Pinene oxide	0.1	tr	tr	-	-	-
1099	1101	Linalool	-	tr	tr	-	tr	tr
1102	1102	6-Methylhepta-3,5-dien-2-one	0.1	-	0.1	-	-	-
1104	1104	Nonanal	-	-	-	-	-	tr
1126	1126	α-Campholenal	tr	-	-	-	-	-
1127	1127	*allo*-Ocimene	tr	tr	tr	tr	-	tr
1130	1130	Limona ketone	tr	tr	tr	-	-	-
1138	1141	(*E*)-Myroxide	-	-	tr	-	-	-
1140	1141	*trans*-Pinocarveol	tr	-	-	-	-	-
1145	1145	*trans*-Verbenol	0.1	-	-	-	-	-
1162	1164	Pinocarvone	tr	-	-	-	-	-
1168	1168	Rosefuran epoxide	tr	-	tr	-	-	-
1180	1180	Terpinen-4-ol	tr	tr	tr	-	tr	tr
1184	1184	*p*-Methylacetophenone	tr	-	-	-	-	-
1184	1187	(3*Z*)-Hexenyl butyrate	-	tr	tr	-	-	tr

1186	1186	*p*-Cymen-8-ol	tr	-	-	-	-	-
1186	1187	Cryptone	-	-	tr	-	-	-
1187	1187	*trans-p*-Mentha-1(7),8-dien-2-ol	-	tr	-	-	-	-
1194	1195	α-Terpineol	0.1	0.1	0.3	-	0.1	tr
1206	1205	Verbenone	tr	-	-	-	-	-
1207	1206	Carveol	-	-	-	-	-	tr
1207	1207	(3*E*)-Octenyl acetate	-	-	tr	-	-	-
1217	1217	*endo*-Fenchyl acetate	tr	-	-	-	-	-
1228	1228	*cis-p*-Mentha-1(7),8-dien-2-ol	-	tr	-	-	-	-
1271	1276	2,3-Pinanediol	tr	-	-	-	-	-
1283	1285	Bornyl acetate	tr	tr	-	-	-	-
1294	1294	*trans*-Pinocarvyl acetate	tr	-	-	-	-	-
1297	1298	Thujyl acetate	0.1	-	-	-	-	-
1345	1346	α-Terpinyl acetate	-	-	0.1	-	-	-
1346	1346	α-Cubebene	-	tr	-	tr	-	0.1
1349	1346	*trans*-Carvyl acetate	-	tr	tr	-	-	-
1357	1361	Neryl acetate	-	0.1	tr	-	0.1	-
1368	1367	Cyclosativene	0.1	0.1	0.1	0.1	0.1	0.2
1370	1370	*iso*-Ledene	0.1	tr	tr	tr	tr	tr
1375	1375	**α-Copaene**	**2.4**	**4.1**	**2.7**	**2.4**	**4.2**	**5.3**
1383	1382	β-Bourbonene	-	-	-	tr	-	tr
1392	1391	1,1,4,7-Tetramethyl-1a,2,3,4,6,7,7a,7b-octahydro-1H-cyclopropa[e]azulene	0.2	tr	tr	0.1	tr	0.1
1403	1405	(*Z*)-Caryophyllene	0.2	0.1	0.2	0.1	tr	0.1
1406	1406	α-Gurjunene	0.3	0.1	0.2	0.2	0.1	0.2
1410	1411	β-Maaliene	tr	tr	tr	tr	-	tr
1413	1412	Longifolene	0.1	tr	tr	0.1	tr	0.1
1415	1414	α-Cedrene	-	-	tr	-	-	-
1420	1417	**(*E*)-β-Caryophyllene**	**13.9**	**20.4**	**21.7**	**30.0**	**24.8**	**27.8**
1426	1430	γ-Maaliene	0.3	0.1	0.1	0.2	0.1	0.1
1430	1431	β-Gurjunene (= Calarene)	0.6	0.1	0.2	0.3	0.1	0.1
1431	1430	β-Copaene	-	-	-	-	-	0.1
1432	1432	*trans*-α-Bergamotene	tr	-	tr	tr	-	-
1433	1435	α-Maaliene	0.2	tr	0.1	0.1	tr	0.1
1439	1439	**Aromadendrene**	**7.5**	**2.9**	**3.0**	**5.9**	**3.0**	**3.5**
1445	1443	Prenyl benzoate	-	-	-	-	-	0.1
1445	1446	Myltayl-4(12)-ene	0.7	0.2	0.2	0.4	0.1	0.2
1448	1447	*iso*-Germacrene D	-	tr	tr	tr	tr	-
1450	1451	*trans*-Muurola-3,5-diene	-	-	-	-	-	tr
1451	1452	(*E*)-β-Farnesene	0.2	tr	0.2	0.1	tr	0.1
1455	1454	**α-Humulene**	**2.7**	**3.0**	**4.0**	**4.3**	**3.6**	**4.7**
1459	1458	*allo*-Aromadendrene	1.5	1.2	1.2	1.3	1.2	1.4
1463	1463	α-Acoradiene	tr	-	tr	-	-	-

1471	1473	*trans*-Cadina-1(6),4-diene	-	0.2	-	0.1	0.3	0.3
1474	1478	γ-Muurolene	0.7	0.6	0.4	0.4	0.6	0.7
1476	1476	γ-Gurjunene	0.1	tr	tr	tr	tr	tr
1479	1482	*ar*-Curcumene	0.2	-	0.2	-	-	-
1486	1489	δ-Selinene	0.2	0.1	0.1	0.1	0.1	0.1
1488	1489	β-Selinene	0.8	0.5	0.4	0.6	0.6	0.7
1490	1491	Viridiflorene	0.5	0.2	0.2	0.4	0.3	0.4
1495	1497	α-Selinene	0.6	0.4	0.2	0.4	0.4	0.5
1497	1497	α-Muurolene	0.3	0.4	0.3	0.3	0.4	0.4
1499	1501	(*Z*)-α-Bisabolene	1.1	0.3	1.5	0.2	0.3	0.4
1502	1506	δ-Amorphene	-	0.1	-	-	0.1	-
1507	1508	β-Bisabolene	2.4	0.3	2.7	0.3	0.3	0.4
1512	1512	γ-Cadinene	0.4	0.3	0.4	0.3	0.3	0.3
1517	1518	δ-Cadinene	0.8	1.9	1.0	1.3	2.4	2.4
1520	1519	*trans*-Calamenene	0.2	1.6	1.4	1.0	1.8	2.3
1526	1528	(*E*)-γ-Bisabolene	-	-	tr	0.1	tr	0.1
1532	1533	*trans*-Cadina-1,4-diene	-	1.4	0.1	0.5	1.8	1.3
1536	1538	α-Cadinene	0.1	0.1	0.1	0.1	tr	0.1
1539	1540	(*E*)-α-Bisabolene	0.2	-	0.2	-	tr	tr
1540	1541	α-Calacorene	0.2	0.3	0.2	0.2	0.2	0.2
1550	1551	(*Z*)-Caryphyllene oxide	0.5	-	0.5	0.2	-	0.2
1554	1548	Caryophyll-5-en-12-al	0.2	0.7	0.2	0.3	0.2	0.1
1556	1555	2*S*,6*S*-2,6,8,8-Tetramethyltricyclo[5.2.2.0(1,6)]undecan-2-ol	0.1	tr	-	-	-	-
1560	1560	**(*E*)-Nerolidol**	**1.4**	**0.1**	**13.7**	**8.6**	**tr**	**7.8**
1561	1560	Germacrene B	3.3	1.2	-	3.0	1.2	-
1569	1568	Maaliol	1.3	0.4	0.5	0.8	0.4	0.6
1571	1571	(3*Z*)-Hexenyl benzoate	-	-	0.2	-	-	-
1572	1569	Longipinocarvone	0.1	0.2	0.1	0.1	-	0.1
1573	1573	Caryophyllene alcohol	0.5	0.7	0.3	-	-	-
1577	1576	Spathulenol	0.1	-	0.3	0.7	0.7	0.8
1583	1587	**Caryophyllene oxide**	**8.1**	**3.7**	**11.4**	**5.7**	**2.4**	**5.3**
1587	1590	**Globulol**	**11.8**	**5.5**	**6.4**	**10.9**	**5.9**	**6.0**
1594	1594	Viridiflorol	1.3	0.6	0.8	1.2	0.7	0.9
1596	1596	Cubeban-11-ol	0.6	0.2	0.3	0.4	0.2	0.2
1598	1596	Humulene epoxide I	0.1	0.1	0.1	0.1	tr	0.1
1604	1605	Ledol	0.4	1.9	1.6	1.3	2.1	2.5
1606	1609	Rosifoliol	1.5	0.4	0.6	0.8	0.5	0.4
1609	1611	Humulene epoxide II	1.0	0.7	1.3	0.7	-	-
1610	1613	Copaborneol	-	-	-	-	1.1	1.5
1614	1614	1,10-di-*epi*-Cubenol	0.1	0.1	0.1	tr	0.2	0.2
1619	1620	*epi*-γ-Eudesmol	0.1	0.2	0.1	0.2	0.1	0.1

1624	1627	Eremoligenol	0.8	0.3	0.3	0.6	0.2	0.4
1625	1624	Muurola-4,10(14)-dien-1β-ol	0.8	1.6	0.8	0.8	-	-
1626	1624	*cis*-Calamenene	-	0.1	-	-	0.3	1.2
1627	1628	1-*epi*-Cubenol	0.7	1.5	1.2	1.1	1.1	1.7
1633	1630	Caryophylla-4(12),8(13)-dien-5α-ol	0.5	0.8	0.7	0.9	1.8	1.2
1637	1636	Caryophylla-4(12),8(13)-dien-5β-ol	1.7	2.6	2.0	2.7	0.8	2.5
1641	1640	τ-Cadinol	0.9	1.7	1.9	1.3	1.7	1.1
1643	1644	τ-Muurolol	0.7	0.6	0.6	0.5	0.7	1.7
1646	1651	α-Muurolol (= δ-Cadinol)	2.1	1.9	1.5	1.6	1.8	2.5
1655	1655	α-Cadinol	1.4	1.1	1.6	1.2	2.2	1.4
1658	1660	*neo*-Intermedeol	0.6	0.4	0.3	0.5	1.4	0.5
1664	1664	*cis*-Calamenen-10-ol	0.1	-	0.1	0.1	0.2	0.2
1668	1669	*epi*-β-Bisabolol	0.2	-	-	-	-	-
1669	1671	14-Hydroxy-9-*epi*-(*E*)-caryophyllene	-	0.3	-	0.4	0.6	0.9
1670	1671	β-Bisabolol	0.4	-	0.7	-	-	-
1672	1672	Cadalene	-	0.1	-	-	0.1	-
1685	1686	*epi*-α-Bisabolol	0.1	-	0.2	tr	-	tr
1687	1688	α-Bisabolol	0.4	0.1	0.6	0.1	0.1	0.1
1700	1700	Heptadecane	-	tr	-	-	-	-
1700	1701	10-*nor*-Calamenen-10-one	tr	-	tr	-	-	-
1713	1714	(2*E*,6*Z*)-Farnesol	-	-	-	-	-	tr
1766	1769	Benzyl benzoate	-	tr	0.1	tr	-	tr
1804	1804	14-Hydroxy-δ-cadinene	tr	-	-	-	-	-
1839	1841	Phytone	tr	tr	tr	-	-	-
2109	2109	Phytol	0.1	tr	0.2	0.1	-	0.4
2500	2500	Pentacosane	-	-	tr	-	-	-
2700	2700	Heptacosane	-	-	tr	-	-	-
		Monoterpene hydrocarbons	14.7	28.0	3.3	0.8	22.3	1.4
		Oxygenated monoterpenoids	0.5	1.2	1.5	0.0	0.8	0.1
		Sesquiterpene hydrocarbons	42.7	42.2	43.3	54.9	49.0	56.2
		Oxygenated sesquiterpenoids	40.7	28.3	50.7	43.6	27.1	41.2
		Diterpenoids	0.1	traces	0.2	0.1	0.0	0.4
		Benzenoid aromatics	traces	traces	0.3	traces	traces	0.1
		Others	0.1	0.0	0.1	0.0	0.0	0.0
		Total identified	98.8	99.7	99.3	99.4	99.2	99.4
